# Comparative Analysis of Deep Learning Architectures for Automatic Tooth Segmentation in Panoramic Dental Radiographs: Balancing Accuracy and Computational Efficiency

**DOI:** 10.3390/diagnostics16020336

**Published:** 2026-01-20

**Authors:** Alperen Yalım, Emre Aytugar, Fahrettin Kalabalık, İsmail Akdağ

**Affiliations:** 1 Department of Oral and Maxillofacial Radiology, Faculty of Dentistry, Izmir Katip Celebi University, Izmir 35640, Turkey; alperen.yalim@ikcu.edu.tr (A.Y.); emre.aytugar@ikcu.edu.tr (E.A.); 2 Department of Oral and Maxillofacial Radiology, Faculty of Dentistry, Sakarya University, Sakarya 54100, Turkey; 3 Department of Electrical and Electronics Engineering, Faculty of Engineering and Architecture, Izmir Katip Celebi University, Izmir 35620, Turkey; ismail.akdag@ikcu.edu.tr

**Keywords:** deep learning, tooth segmentation, panoramic radiography, computational efficiency, dental imaging

## Abstract

**Background/Objectives:** This study provides a systematic benchmark of U-Net–based deep learning models for automatic tooth segmentation in panoramic dental radiographs, with a specific focus on how segmentation accuracy changes as computational cost increases across different encoder backbones. **Methods:** U-Net models with ResNet, EfficientNet, DenseNet, and MobileNetV3-Small encoder families pretrained on ImageNet were evaluated on the publicly available Tufts Dental Database (1000 panoramic radiographs) using a five-fold cross-validation strategy. Segmentation performance was quantified using the Dice coefficient and Intersection over Union (IoU), while computational efficiency was characterized by parameter count and floating-point operations reported as GFLOPs per image. Statistical comparisons were conducted using the Friedman test followed by Nemenyi-corrected post hoc analyses (p<0.05). **Results:** The overall segmentation quality was consistently high, clustering within a narrow range (Dice: 0.9168–0.9259). This suggests diminishing returns as the backbone complexity increases. EfficientNet-B7 achieved the highest nominal accuracy (Dice: 0.9259 ± 0.0007; IoU: 0.8621 ± 0.0013); however, the differences in Dice score between EfficientNet-B0, B4 and B7 were not statistically significant (p>0.05). In contrast, computational demands varied substantially (2.9–67.2 million parameters; 4.93–40.8 GFLOPs). EfficientNet-B0 provided an accurate and efficient operating point (Dice: 0.9244 ± 0.0011) at low computational cost (5.98 GFLOPs). In contrast, MobileNetV3-Small offered the lowest computational cost (4.93 GFLOPs; 2.9 million parameters), but also the lowest Dice score (0.9168 ± 0.0031). Compared with heavier ResNet and DenseNet variants, EfficientNet-B0 achieved competitive accuracy with a markedly lower computational footprint. **Conclusions:** The findings show that larger models do not always perform better and that models with increased performance may not necessarily yield meaningful gains. It should be noted that the findings are limited to the task of tooth segmentation; different findings may be obtained for different tasks. Among the models evaluated for tooth segmentation, EfficientNet-B0 stands out as the most practical option, maintaining near-saturated accuracy levels while keeping model size and computational cost low.

## 1. Introduction

In modern clinical dentistry, the use of radiographic imaging is crucial for the evaluation of anatomy, diseases, and treatment planning [1]. Recent advances in dental image analysis have taken place due to deep learning (DL) methods and, more particularly, methods based on convolutional neural networks (CNN) [2]. Models that use convolutional neural networks (CNNs) have shown high accuracy in several clinical tasks, including the detection of dental caries [3], anatomical segmentation [4], periodontal status assessment [5], and automated analysis of oral lesions [6]. They also show good results in various imaging modalities such as panoramic radiographs, cone beam computed tomography (CBCT), and intraoral images [7].

Although these advancements have occurred, it is still challenging to translate DL systems from controlled research settings to routine clinical workflows due to computational limitations [8]. According to the sustainable paradigm artificial intelligence (AI), it is important to have not only high accuracy but also low computational cost and compatibility with cheap hardware [9]. Consequently, many of the applications of modern architectures’ in the clinic experience limitations due to high processing demand, excessive memory usage, or low inference speed for real-time applications [8,10,11].

The computational footprint of deep learning models is typically characterized using two fundamental metrics: the number of trainable parameters and floating-point operations (FLOPs) [12]. The number of parameters directly affects the model’s storage requirements and memory usage; therefore, it is a critical variable for integration into resource-constrained clinical hardware [13,14]. Furthermore, models with high architectural capacity may be more prone to overfitting when not supported by sufficiently large labeled datasets [14,15]. In contrast, FLOPs quantify a network’s theoretical arithmetic computational complexity [16]. On a fixed deployment platform, increasing FLOPs values are expected to lead to longer processing times and higher computation-related energy consumption [16,17]; however, real-world efficiency may also be influenced by memory access patterns and implementation details [18,19,20].

Current methodological guidelines emphasize the need for systematic, transparent, and hypothesis-driven approaches in the design and reporting of DL studies in dentistry, underscoring the importance of deliberate choices regarding model architecture, architectural complexity, and weight initialization strategies [21,22]. Model architecture builds upon the foundational concept of artificial neurons introduced by McCulloch and Pitts (1943) [23], in which nonlinear computational units are organized into layered structures that progressively transform input information. In the context of segmentation, this architectural component corresponds to the backbone, which extracts multi-scale and semantically rich feature representations from the input image, forming the foundation upon which pixel-wise segmentation decisions are generated [24].

In the literature, most deep learning-based tooth segmentation studies on panoramic radiographs report performance predominantly through accuracy-based metrics (e.g., Dice coefficient, IoU, and F1 score), while efficiency and computational cost are often excluded [25,26,27]. For instance, Ma et al. proposed a multi-feature coordinate position learning–based approach for panoramic tooth segmentation; however, they did not explicitly report hardware-agnostic indicators of model complexity such as parameter count or FLOPs/GFLOPs ($10^{9}$ FLOPs) in a format suitable for comparative assessment [28]. Similarly, although Hou et al. described multiple architectural modules within Teeth U-Net and specified the experimental platform, they did not quantitatively provide computational-cost indicators such as model size and FLOPs [29]. In contrast, Lin et al. framed panoramic segmentation as an edge-device deployment problem, reported the number of parameters, discussed the practical implications of computational load, and emphasized that such efficiency indicators are often missing in the segmentation literature [30]. Although Khaldi et al. and Ma et al. partially addressed this gap by reporting hardware-independent metrics such as the number of parameters and FLOPs/GFLOPs in their studies, domain shift arising from different data domains and acquisition conditions, along with input resolution, preprocessing pipelines, and variability in experimental setups, complicate direct benchmarking between studies [31,32]. Meanwhile, some studies report efficiency using time-based metrics such as runtime/latency or inference speed/FPS [33,34]. In addition, time-based efficiency metrics are substantially influenced by differences in the hardware used (e.g., GPU/CPU), the software stack, and the measurement protocol, which can complicate direct cross-study comparisons [35,36]. Motivated by these gaps, the present study aims to establish a transparent accuracy–efficiency trade-off across backbone architectures by reporting hardware-agnostic efficiency indicators, namely parameter count and GFLOPs.

To analyse this trade-off at the backbone level, we considered four families of backbone architecture that reflect different design philosophies. ResNet improves the stability of optimization in deep networks through residual connections [37], EfficientNet targets efficiency via compound scaling [38], and DenseNet promotes feature reuse through dense connectivity [39]. Additionally, we included the MobileNet family as a lightweight paradigm representative of depthwise separable convolutions, which aims to reduce computational and memory demands while maintaining competitive representational capacity [40]. Despite these architectures being widely used in general computer vision, there are few comprehensive dental imaging studies that compare them side by side in terms of both segmentation accuracy and computational efficiency [21,41,42,43]. This leaves a relatively fragmented comparative evidence base in this field.

Accordingly, this study adopts the widely used U-Net architecture as a baseline for biomedical image segmentation [44,45] and constructs a modular framework by replacing its encoder module with ImageNet [46] pretrained models from the ResNet, EfficientNet, DenseNet and MobileNet families. This approach allows for a direct assessment of the effects of architectural variations on tooth segmentation. The primary objective of this work is to provide a systematic comparison of these encoders regarding accuracy and computational efficiency, thereby contributing to the identification of lightweight, high-performance models that represent promising candidates for future clinical deployment, pending further validation and real-time performance assessment.

## 2. Materials and Methods

### 2.1. Dataset and Experimental Setup

The Tufts Dental Database [47] was utilized as a benchmarking resource in dental radiography to evaluate the performance of deep learning–based segmentation models. The dataset comprises 1000 panoramic radiographs acquired in clinical settings. Radiographs were randomly selected from the Tufts University electronic patient database (axiUm) between 2014 and 2016 and were acquired using OP100 Orthopantomograph (Instrumentarium Dental, Tuusula, Finland) and Planmeca ProMax 2D (Planmeca, Helsinki, Finland) with automatic exposure control. The inclusion criterion was optimal diagnostic image quality with minimal or no technical errors. Images were provided as de-identified TIFF/JPEG files.

The dataset includes panoramic images from patients with and without tooth loss, contains common restorative/prosthetic findings (e.g., amalgam/composite restorations and crowns), and includes both pediatric and adult radiographs, with pediatric images also used in the present study. All available samples were included in the analysis without preselection. The segmentation task aimed to delineate “tooth” regions from the “background”. Accordingly, binary masks defining tooth boundaries provided by expert annotators served as the ground truth. Because the public release does not include patient-level demographics or detailed acquisition metadata beyond the unit models, stratified analyses by age or device subgroups were not feasible.

### 2.2. Data Preprocessing

Panoramic radiographs inherently present a wide rectangular field of view; therefore, all images and corresponding masks were rescaled from 1615 × 840 to 512×256 pixels while preserving the original aspect ratio (≈2:1). This choice reduces computational cost and memory usage while minimizing geometric distortion that may arise from forcing a square input. Similar 2:1 preprocessing resolutions (e.g., 512 × 256) have also been adopted in prior panoramic deep-learning studies [48,49].

Geometric data augmentation techniques were employed during the training phase to improve the generalization capability of the models and prevent overfitting. Specifically, random horizontal and vertical flips were applied to the original images and their corresponding masks. Through these augmentation procedures, the total dataset size was expanded from 1000 to 3000 images, ensuring a more robust training process.

### 2.3. Architecture and Encoder Selection

In our study, we selected the U-Net architecture as the core model, as it is widely used as a baseline in biomedical image segmentation [50]. Due to the limited labeled training data, we utilized transfer learning by first initializing the encoder weights with ImageNet pre-trained weights. This allowed for the fine-tuning of the network on our dataset to enable faster convergence and better generalization. Previous studies on medical imaging show that sufficiently fine-tuned pretrained CNNs perform equally or better than those trained from scratch with additional benefit of robustness when training data is scarce [51,52].

**Figure 1:**
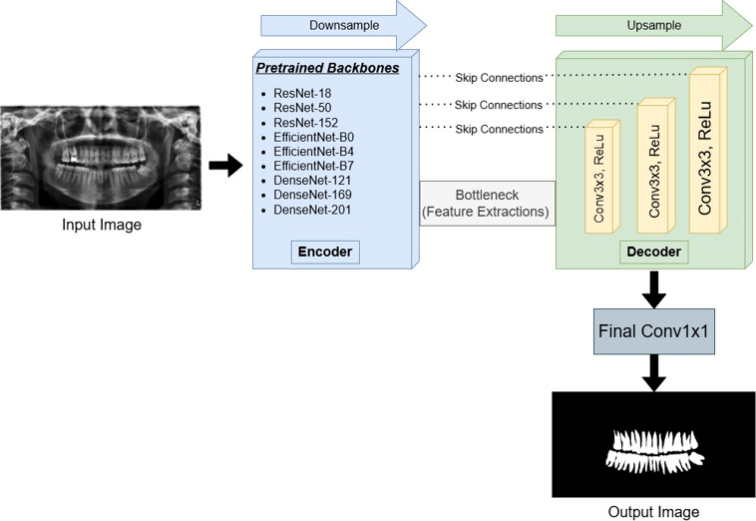
Schematic overview of the proposed U-Net-based architecture for automatic teeth segmentation. The network utilizes pretrained backbones (ResNet, EfficientNet, and DenseNet series) as the encoder for feature extraction. The decoder path upsamples the feature maps, fusing them with high-resolution details from the encoder via skip connections. The final 1 × 1 convolution layer generates the binary segmentation mask.

As shown in Figure 1, the standard U-Net encoder was replaced with modern CNN backbones, while the conventional U-Net decoder design was retained. The models were implemented using the segmentation models pytorch library [53], which provides a simple way to couple various pretrained encoders with a U-Net–style decoder. Skip connections forward multi-scale encoder feature maps to the decoder to preserve spatial detail. During decoding, feature maps are progressively upsampled by a factor of two and fused with the corresponding encoder features, allowing the network to recover fine-grained structure. Finally, a convolution layer produces the pixel-wise segmentation mask.

#### 2.3.1. ResNet

**Figure 2:**
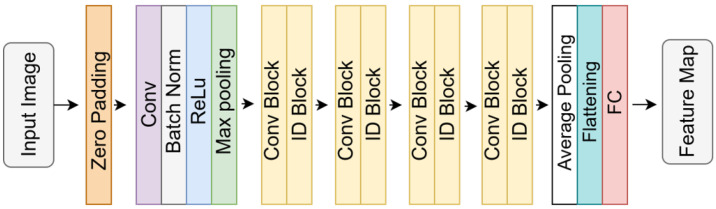
Block diagram of the ResNet architecture, illustrating the “ID Blocks” with residual connections to facilitate gradient flow.

The ResNet architecture, developed to overcome the problem of vanishing gradients in deep neural networks, is widely favored in medical image analysis due to its stable learning dynamics and its ability to mitigate performance degradation in deeper structures [37,54]. As illustrated in Figure 2, the fundamental units of the architecture, identity blocks, contain skip connections that directly propagate the input information to deeper layers. These connections facilitate the transfer of low-level features without degradation, which is particularly beneficial for dental radiographs that are often characterized by low contrast and complex visual patterns. ResNet-(18, 50 and 152) representing shallower and deeper variants, respectively, were evaluated in this study.

#### 2.3.2. EfficientNet

**Figure 3:**
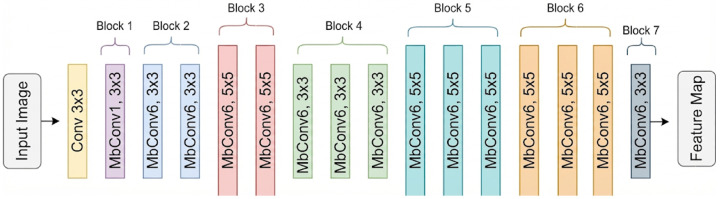
Block diagram of the EfficientNet architecture, featuring a sequence of “MBConv” blocks optimized for parameter efficiency.

EfficientNet is a modern CNN architecture that employs a compound coefficient scaling strategy to simultaneously optimize the depth, width, and input resolution of the network [38]. This approach aims to enhance performance while maintaining the efficiency of the parameters. As depicted in Figure 3, EfficientNet replaces conventional convolutional layers with MBConv blocks (Mobile Inverted Bottleneck Convolution). Through bottleneck structures and separable convolutions in depth, MBConv blocks reduce computational cost while increasing representational capacity. In addition, squeeze-and-excitation operations within these blocks improve the transformation of channel-wise information. EfficientNet-(B0, B4, and B7) representing different scaling configurations were included in the comparative analysis.

#### 2.3.3. DenseNet

**Figure 4:**
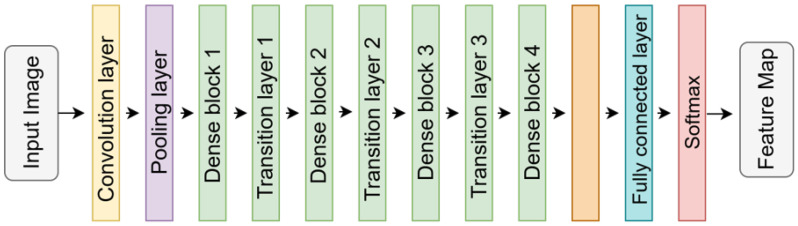
Block diagram of the DenseNet architecture, showcasing the “Dense Blocks” connected via “Transition Layers” for feature reuse.

The DenseNet architecture introduces a densely connected design where each layer receives inputs from all preceding layers [39]. This configuration aims to minimize information loss as network depth increases and facilitate gradient flow. As shown in Figure 4, the structure comprises dense blocks and transition layers. Dense blocks enrich information flow by concatenating inter-layer outputs along the channel dimension, while transition layers apply compression to reduce channel count and spatial resolution, thereby improving parameter efficiency. This architecture ensures the simultaneous availability of low- and high-level features, maximizing feature reuse. DenseNet-(121, 169 and 201) differing in depth and parameter density, were evaluated.

#### 2.3.4. MobileNet

**Figure 5:**
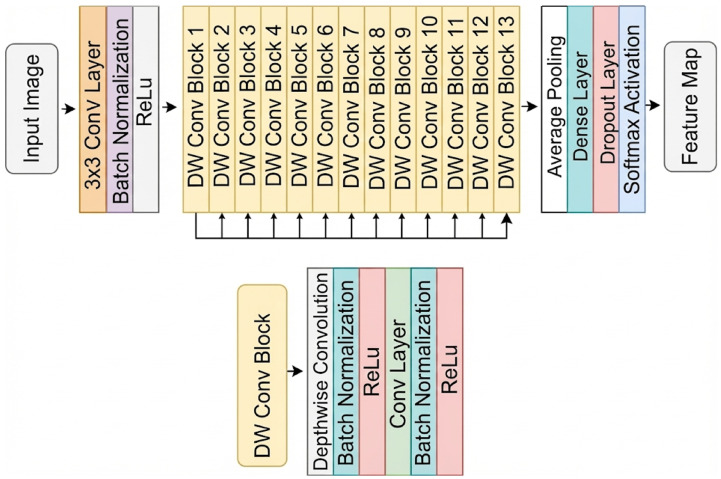
Block diagram of the MobileNet architecture. Feature extraction is performed through a sequence of DW Conv Blocks (1–13), whose internal structure is summarized in the inset. The network is finalized with global pooling and a lightweight head.

The MobileNet family follows an efficiency-oriented design tailored for resource-constrained inference by using depthwise separable convolutions as its primary building principle [40]. This design substantially reduces computation by decoupling spatial filtering from channel mixing, enabling compact networks that still preserve effective feature extraction. As illustrated in Figure 5, the architecture consists of an initial convolutional stem followed by a sequence of DW Conv Blocks, which serve as lightweight modules for hierarchical representation learning. In practice, these blocks are commonly organized in bottleneck-style configurations and may incorporate channel-attention mechanisms to strengthen informative feature channels with minimal overhead. In this study, MobileNetV3-Small, a compact member of the MobileNet family, was evaluated as the encoder backbone within the U-Net framework.

### 2.4. Model Training and Implementation Details

Model development and training were conducted using the Python programming language (version 3.12.10) and the PyTorch library(version 2.4.1). For consistency and reproducibility, all experiments, including statistical analyses, were performed in the same Python environment (v3.12.10). All computations were performed using an NVIDIA GeForce RTX 4060 Ti GPU with 16 GB of VRAM. We used the AdamW optimizer because of its decoupled weight decay formulation, which separates weight decay from adaptive gradient updates. This formulation has been reported to improve optimization stability and generalization under the right hyperparameter settings [55]. The initial learning rate was set to $1\times 10^{-3}$, with a batch size of 16, to maximize GPU utilization and training stability. No learning rate scheduler was applied, and the learning rate was kept constant throughout training.

We used 5-fold cross-validation in order to ensure robustness of results. The dataset was divided into five equal subsets; in each fold, one subset (20%) was taken as the validation/test and the remaining four (80%) were used for training. This structure ensured that all images appeared exactly once in the validation set. The final performance metric is the average of the 5 folds. With respect to training dynamics, we trained the models for a fixed period of 25 epochs without early stopping while fine-tuning all encoder weights end-to-end. We fixed random seeds across Python, NumPy and Pytorch to ensure reproducibility.

In segmentation, we used BCE and Dice loss in a hybrid loss function. This formulation utilizes BCE, which assesses pixel-level sensitivity, together with Dice loss, which combats class imbalance, to correctly define anatomical boundaries.

### 2.5. Evaluation Metrics

Model performance was comprehensively evaluated across two primary axes: segmentation accuracy and computational efficiency.

#### 2.5.1. Segmentation Performance Metrics

Four fundamental metrics assessed segmentation performance:

- Dice Similarity Coefficient (DSC): The primary similarity metric quantifying the overlap between the predicted mask (*P*) and ground truth (*G*):
  (1) $$DSC=\frac{2|P\cap G|}{\left|P|+|G\right|}$$
- Jaccard Index (Intersection over Union, IoU): The ratio of the intersection area to the union area, which is more sensitive to errors than the Dice coefficient:
  (2) $$IoU=\frac{\left|P\cap G\right|}{\left|P\cup G\right|}$$
- Precision: Indicates the accuracy of positive predictions; low values suggest over-segmentation:
  (3) $$Precision=\frac{TP}{TP+FP}$$
- Recall (Sensitivity): Indicates the proportion of actual positive pixels correctly identified; low values suggest under-segmentation:
  (4) $$Recall=\frac{TP}{TP+FN}$$

#### 2.5.2. Computational Efficiency Metrics

Resource consumption and architectural complexity were assessed using two fundamental technical metrics. The first metric, the number of trainable parameters, represents the total count of weights and biases defining a model’s learning capacity and directly reflects memory requirements (RAM/VRAM) as well as disk storage footprint. The second metric, floating-point operations (FLOPs), provides a hardware-independent theoretical measure of computational complexity by estimating the number of floating-point operations required to process a single image (i.e., one forward pass). In this study, computational complexity is reported in GFLOPs per image. Although neither metric fully captures real-time inference latency on specific clinical hardware, their combined use offers a standardized and objective proxy for comparing the relative computational demands of candidate models.

### 2.6. Statistical Analysis

All statistical analyses were performed using Python (version 3.12.10). The Friedman test for repeated dependent measurements and subsequent post hoc comparisons were conducted using the SciPy library(1.16.3). A nonparametric statistical framework was adopted to evaluate performance differences across the fivefold cross-validation, as all architectures were tested on identical validation folds. Fold-level Dice similarity coefficients were used for all analyses, with statistical significance set at p<0.05.

## 3. Results

As shown in Table 1, all U-Net–based encoder architectures achieved consistently high segmentation performance on panoramic radiographs. Dice coefficients ranged from 0.9168 to 0.9259 across the evaluated models, with most architectures clustering around ∼0.92. EfficientNet-B7 achieved the highest Dice and IoU scores (Dice: 0.9259±0.0007; IoU: 0.8621±0.0013), followed by EfficientNet-B4 and EfficientNet-B0.

Precision and recall values were also generally high. The highest precision was observed for EfficientNet-B4 (0.9271±0.0029). Recall values showed limited variation across models and were reported in the range of 0.9185–0.9252. For MobileNetV3Small, the results were Dice: 0.9168 ± 0.0031; IoU: 0.8464 ± 0.0053; Precision: 0.9154 ± 0.0098; Recall: 0.9184 ± 0.0081.

In terms of model complexity and computational cost, the number of parameters and per-image GFLOPs differed substantially across encoders. Parameter counts ranged from 2.9 M to 67.2 M, while the computed per-image computational cost ranged from 4.93 to 40.80 GFLOPs.

**Figure 6:**
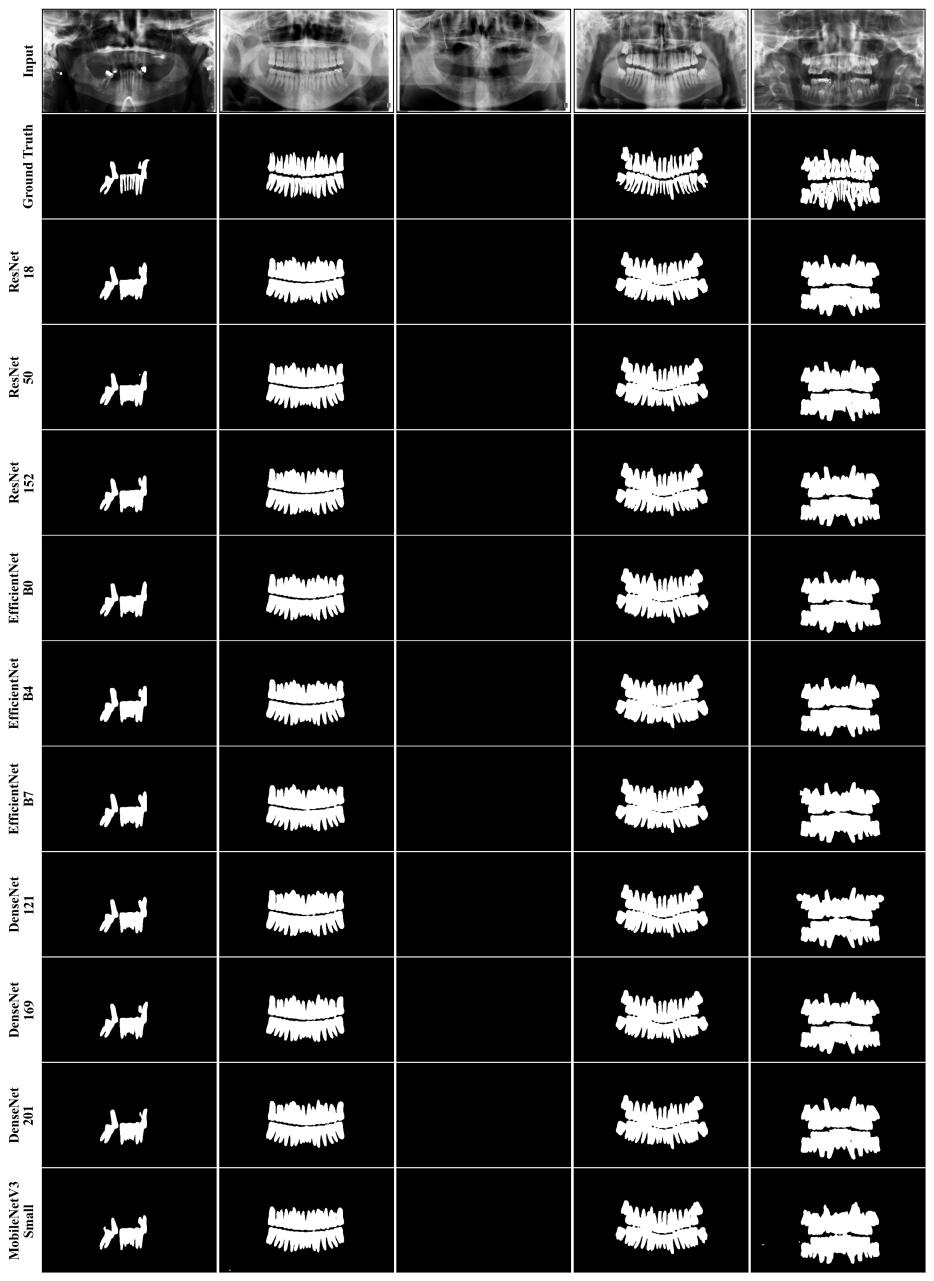
Qualitative comparison of tooth segmentation results obtained with U-Net–based encoder backbones on representative test samples. The first row shows the input panoramic radiographs, the second row shows the expert-annotated ground truth (GT), and subsequent rows show the predicted binary tooth masks generated by each backbone. Columns represent distinct clinical/anatomical scenarios: partial tooth loss (column 1), no tooth loss (column 2), edentulous (column 3), no tooth loss with impacted third molars (column 4), and mixed dentition (column 5).

The tooth masks predicted by each backbone are qualitatively compared in Figure 6, which shows that these masks have a broadly similar overall morphology. The selected cases are meant to reflect the clinical variability of the dataset: partial tooth loss (column 1), no tooth loss (column 2), edentulous (column 3) and no tooth loss with impacted third molars (column 4), and mixed dentition (column 5). In the majority of samples, the tooth regions are reliably delineated with maintained interproximal separation and cervical contour and impacted third molars are captured as well (column 4). The differences that do occur are on small and difficult areas, for example, areas with very thin apical contours or small disconnected fragments where some predictions slightly undersegment the area of interest or are slightly beyond. In fifth column, the apical boundary is more irregular in MobileNetV3-Small, and 3rd column is a control case, with all models giving the correct empty masks. Overall, the qualitative differences did not follow a consistent trend with model complexity; larger backbones do not consistently give better mask fidelity, and the differences are subtle, localized and are mostly sample dependent.

### 3.1. Statistical Comparison of Model Performances

The fold-level Dice coefficients for each encoder architecture are presented in Table 2. The comparative analysis of these ten deep learning models was conducted using the Friedman test followed by the Nemenyi post hoc test. The Friedman test indicated a statistically significant difference in model performance ($\chi^{2}=37.93$, p<0.001). Importantly, this overall significance indicates that at least one model differs from the others and does not imply statistically significant differences for all pairwise model comparisons. According to the mean rankings listed in Table 3, EfficientNet-B7 achieved the best performance (Mean Rank: 1.60), while MobileNetV3Small ranked the lowest (Mean Rank: 10.00); the ResNet architectures (ResNet18 and ResNet152) also ranked among the lowest (Mean Rank: 8.00). Given the narrow fold-level Dice range observed across models (approximately 0.914–0.927), the mean-rank ordering should be interpreted alongside the post hoc pairwise results.

The pairwise comparison results, detailed in Table 4, show statistically significant differences in a limited subset of comparisons. Specifically, EfficientNet-B7 showed statistically significant improvements over ResNet18 (p=0.029) and ResNet152 (p=0.029), and also differed significantly from MobileNetV3Small (p<0.001). In addition, EfficientNet-B0 (p=0.010), EfficientNet-B4 (p=0.007), and DenseNet169 (p=0.040) showed statistically significant differences compared to MobileNetV3Small. Other comparisons did not yield statistically significant differences (p>0.05). Therefore, while the Friedman test supports an overall difference among models, the Nemenyi-corrected post hoc analysis indicates that statistically significant pairwise differences are limited to a small subset of comparisons (Table 5).

### 3.2. Computational Efficiency and Performance Balance

**Figure 7:**
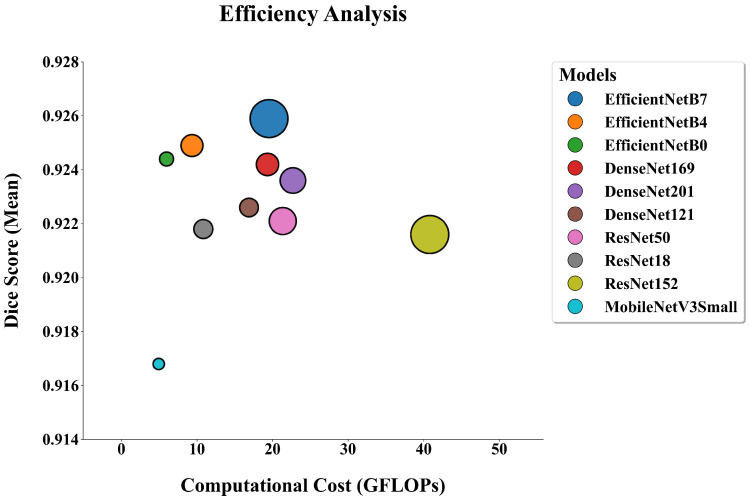
Efficiency plot illustrating the trade-off between segmentation accuracy (Dice coefficient, y-axis) and computational cost (GFLOPs per image, x-axis). Bubble size represents the number of trainable parameters.

Figure 7 summarizes the balance between segmentation accuracy and computational cost. In this efficiency plot, a more desirable operating point is typically located toward the upper-left region, where higher Dice is achieved with lower per-image GFLOPs (with bubble size reflecting parameter count). The results suggest that reducing computational cost does not necessarily lead to a proportional decrease in accuracy; however, extremely lightweight backbones may exhibit a clearer trade-off. Specifically, MobileNetV3Small has the lowest computational cost (4.93 GFLOPs, 2.9 M parameters) but is also associated with the lowest Dice (0.9168). In contrast, EfficientNet-B0 attains a higher Dice score (0.9244) while still requiring low computation (5.98 GFLOPs, 6.3 M parameters). Compared with larger EfficientNet variants, EfficientNet-B0 requires fewer GFLOPs than EfficientNet-B4 (9.34 GFLOPs) and EfficientNet-B7 (19.53 GFLOPs), and the Dice differences among EfficientNet-B0/B4/B7 were not statistically significant (p>0.05). Overall, the distribution in Figure 7 indicates that EfficientNet-B0 may represent a favorable accuracy–efficiency operating point within the evaluated backbones.

### 3.3. Use of Generative AI Tools

Generative AI tools were used to improve English language, grammar, and readability of the manuscript, and during code development to assist with writing and refining scripts used for aggregating the reported metrics and generating visualizations (tables/plots) for the Section 3.

## 4. Discussion

This study compared various deep learning architectures for the automatic segmentation of teeth in panoramic radiographs, evaluating both the accuracy of the segmentation and the computational efficiency. The task was designed as a binary segmentation problem (tooth vs background) to control for encoder-backbone effects under a fixed label definition, thereby enabling direct benchmarking. The binary tooth masks generated can be used as a standard initial output to aid downstream applications such as instance-level tooth numbering or multiclass segmentation of dental structures and diseases. All evaluated models achieved high Dice scores within a narrow range, consistent with previous studies reporting strong performance of U-Net–based architectures for panoramic tooth segmentation [56,57,58]. Nevertheless, as architectural complexity increased, accuracy gains remained marginal, suggesting near-saturation within this benchmark setting and a trend consistent with diminishing returns for this dataset and task. Furthermore, because Dice values clustered within a narrow range, statistically significant differences in ranking may correspond to modest performance changes rather than universally clinically meaningful improvements. Given that the observed differences were modest and performance can be sensitive to data partitioning, the findings were supported by five-fold cross-validation together with an appropriate statistical evaluation to improve the robustness of the comparative analysis [45,59,60].

This observation is in line with the law of diminishing returns frequently discussed in the deep learning literature [61,62,63,64,65,66]. The relationship between network complexity and accuracy followed a nonlinear, logarithmic pattern, suggesting that substantial increases in model size resulted in only limited performance improvements [38,67]. Across encoder backbones, the observed Dice differences were relatively small compared with the substantial variation in computational footprint (GFLOPs and parameter count). This pattern may be consistent with an accuracy–complexity regime in which additional model capacity yields limited gains while computational requirements increase markedly. Therefore, reporting efficiency metrics alongside segmentation performance can help contextualize whether higher-capacity designs provide practically meaningful benefits beyond marginal improvements.

In this study, the evaluation of deep learning architectures was not limited solely to segmentation accuracy; instead, an analysis of computational efficiency was incorporated into the evaluation framework. Although inference latency is often reported as a practical performance metric, it is considered structurally unstable for architectural comparisons due to its strong dependence on hardware configurations, memory bandwidth bottlenecks, and software stack optimizations [68]. Moreover, recent studies have demonstrated that a linear relationship does not always exist between arithmetic complexity and actual runtime performance in convolutional neural networks, primarily due to an “efficiency gap” arising from memory access costs [69].

To mitigate these hardware-specific biases, the present study adopted the number of floating-point operations (FLOPs) as the primary indicator of theoretical computational workload. In the literature, FLOPs are widely recognized as a hardware-agnostic complexity metric that enables objective and fair comparisons among deep learning models with differing architectural design principles [70,71,72]. This perspective is also consistent with recent and comprehensive reviews on model efficiency, which emphasize that the practical implementationability of deep learning systems should be assessed not only in terms of accuracy but also considering the overall “model footprint,” which includes factors such as parameter count and computational cost [73].

Figure 7 shows that the accuracy–efficiency trade-off is more pronounced at the extreme low-compute end. MobileNetV3-Small achieved the lowest Dice score (0.9168) but also the lowest computational cost among the evaluated backbones (4.93 GFLOPs per image; 2.9 M parameters). Post hoc comparisons indicated statistically significant differences between MobileNetV3-Small and EfficientNet-B0, EfficientNet-B4, EfficientNet-B7, and DenseNet-169 (p<0.05), suggesting that the reduction in Dice is unlikely to be a chance fluctuation. The qualitative examples in Figure 6 are consistent with these quantitative outcomes. Rather than implying a general pattern, they illustrate that in specific challenging regions MobileNetV3-Small may produce more variable boundary delineation; notably, the irregular apical boundary in column 5 provides an example of such a case. The importance of this result should neither be overstated nor downplayed, and it should not be interpreted as indicating clinical “superiority” or “inferiority” in all cases. Ultimately, clinical relevance depends on the intended use case and on how the segmentation output is incorporated into the clinical decision pathway.

Besides computational tradeoffs, generalizability is affected by domain shift and anatomical variability. The dataset is representative of what has routinely been seen in clinics. It has a variety of dentition status that is with and without tooth loss, and the appearance of restorations and prostheses on the pediatric and adult radiographs. Nevertheless, the quality of panoramic images may differ significantly across devices and acquisition conditions (e.g., exposure and contrast adjustments, sharpness, positioning(bias) artifacts and metal-related intensity differences) and the public release does not include patient-level demographics and acquisition metadata to quantify them in stratified subgroups. As such, performance under out-of-domain conditions may not align with the benchmark results reported here, highlighting the importance of performing cross-device, multi-center evaluation and if needed domain adaptation or calibration.

In light of these results, and considering the trade-off between accuracy and efficiency, we found EfficientNet-B0 to be an appealing architecture, as it achieved high segmentation accuracy despite being lightweight and requiring relatively few FLOPs. A review of the literature reveals that models based on EfficientNet offer a more favorable trade-off between accuracy and efficiency compared to other families of architectures [43,74,75,76,77]. According to several studies in dental image analysis, EfficientNet-based models can achieve high and stable results in classification and segmentation tasks [26,43,59,78,79,80]. Studies with limited data have reported that the EfficientNet family obtains high parameter efficiency through compound scaling that balances network depth, width, and input resolution [38,80]. In line with these reports, the literature also includes radiographic dental diagnosis classification studies in which EfficientNet-B0 can achieve higher accuracy than the more complex ResNet and DenseNet variants [79,80]. Reports also suggest that using EfficientNet encoders with U-Net architectures significantly improves the Dice and IoU of anatomical structures for segmentation tasks [26,59]. Similarly, our findings indicate that the EfficientNet-B0 model achieves high segmentation accuracy despite having a limited number of parameters and FLOPs. This indicates that computational complexity can be reduced without sacrificing accuracy. Therefore, EfficientNet-based encoders are strong candidates for dental radiology use cases where efficiency is a key requirement. Lower parameter counts and GFLOPs correspond to a smaller memory footprint and reduced compute requirements, which may enable use in chairside software, on-device inference, or resource-constrained clinical settings.

Deeper and more complex architectures may offer advantages in tasks involving greater data heterogeneity, anatomical variability or more challenging segmentation objectives. In contrast, high accuracy can often be achieved using more lightweight architectural designs in well-defined and structurally consistent tasks such as panoramic tooth segmentation. In this context, the limited additional benefit provided by increased architectural complexity should be interpreted as a consequence of the characteristics of the task, rather than as a universally applicable advantage.

## 5. Limitations

This study naturally has some limitations. First, models were trained and evaluated using panoramic radiographs from a single academic center (Tufts Dental Database), which may limit generalizability to other populations and imaging protocols. Second, the comparison was restricted to ResNet, EfficientNet, DenseNet and MobileNet architectures; transformer-based or hybrid architectures were not explored. Third, although FLOPs were adopted as a hardware-agnostic measure of computational complexity, this metric represents a theoretical estimate and does not fully capture real-world runtime behavior under clinical deployment conditions. Finally, the models were not integrated into an actual clinical workflow, and prospective clinical validation was beyond the scope of the present study.

## 6. Conclusions

This study systematically evaluated U-Net–based encoder backbones for automatic tooth segmentation in panoramic radiographs while jointly considering segmentation accuracy, computational efficiency, and statistical validation. In this benchmark, we see that all models perform well in segmentation. Further, increasing the complexity of the backbone models only yields small improvements in accuracy. If accuracy and efficiency are considered jointly, EfficientNet-B0 offers a favorable trade-off between segmentation performance and computational demand. Nevertheless, it is essential to consider that EfficientNet-B0 did not achieve the highest Dice score in the study (e.g., EfficientNet-B7 had the highest mean Dice). Consequently, this observation should be interpreted with caution in light of the limitations of the study and the end-use clinical application. To enhance generalizability assessment during the domain shift, these findings should be validated across devices and clinical centers.

## Figures and Tables

**Table 1:** Model performance (Dice, IoU, precision, and recall; mean ± SD), model complexity (parameters, M), and computational cost (GFLOPs) of U-Net–based encoder backbones for automatic tooth segmentation on panoramic radiographs.

Model	Dice (Mean ± SD)	IoU (Mean ± SD)	Precision (Mean ± SD)	Recall (Mean ± SD)	Params (M)	GFLOPs
EfficientNetB7	0.9259 ± 0.0007	0.8621 ± 0.0013	0.9268 ± 0.0047	0.9252 ± 0.0048	67.1	19.53
EfficientNetB4	0.9249 ± 0.0011	0.8604 ± 0.0020	0.9271 ± 0.0029	0.9230 ± 0.0046	20.2	9.34
EfficientNetB0	0.9244 ± 0.0011	0.8596 ± 0.0019	0.9241 ± 0.0044	0.9251 ± 0.0067	6.3	5.98
DenseNet169	0.9242 ± 0.0016	0.8592 ± 0.0028	0.9260 ± 0.0063	0.9227 ± 0.0075	21.2	19.33
DenseNet201	0.9236 ± 0.0024	0.8581 ± 0.0042	0.9243 ± 0.0026	0.9231 ± 0.0062	28.6	22.70
DenseNet121	0.9226 ± 0.0007	0.8565 ± 0.0013	0.9237 ± 0.0070	0.9218 ± 0.0057	13.6	16.89
ResNet50	0.9221 ± 0.0021	0.8556 ± 0.0036	0.9211 ± 0.0058	0.9234 ± 0.0081	32.5	21.35
ResNet18	0.9218 ± 0.0020	0.8550 ± 0.0034	0.9253 ± 0.0017	0.9185 ± 0.0049	14.3	10.83
ResNet152	0.9216 ± 0.0014	0.8547 ± 0.0025	0.9192 ± 0.0038	0.9242 ± 0.0050	67.2	40.80
MobileNetV3Small	0.9168 ± 0.0031	0.8464 ± 0.0053	0.9154 ± 0.0098	0.9184 ± 0.0081	2.9	4.93

**Table 2:** Fold-level Dice coefficients for each encoder architecture.

Model	Fold 1	Fold 2	Fold 3	Fold 4	Fold 5
ResNet18	0.9191	0.9215	0.9246	0.9220	0.9218
ResNet50	0.9201	0.9237	0.9243	0.9196	0.9227
ResNet152	0.9194	0.9220	0.9232	0.9212	0.9222
EfficientNet-B0	0.9233	0.9243	0.9263	0.9244	0.9240
EfficientNet-B4	0.9232	0.9260	0.9259	0.9244	0.9251
EfficientNet-B7	0.9250	0.9269	0.9263	0.9254	0.9258
DenseNet121	0.9214	0.9230	0.9229	0.9226	0.9233
DenseNet169	0.9232	0.9238	0.9270	0.9230	0.9242
DenseNet201	0.9201	0.9241	0.9270	0.9235	0.9233
MobileNetV3Small	0.9177	0.9155	0.9217	0.9139	0.9150

**Table 3:** Mean ranks obtained from the Friedman test.

Model	Mean Rank
EfficientNet-B7	1.60
EfficientNet-B4	2.80
EfficientNet-B0	3.00
DenseNet169	3.80
DenseNet201	4.20
DenseNet121	6.60
ResNet50	7.00
ResNet152	8.00
ResNet18	8.00
MobileNetV3Small	10.00

**Table 4:** Nemenyi post hoc analysis results (p≤0.05). The column numbers (1–10) correspond to the model indices listed in the first column. * indicates statistically significant pairwise differences after Nemenyi correction.

No	Model	1	2	3	4	5	6	7	8	9	10
1	ResNet18	–									
2	ResNet50	1.000	–								
3	ResNet152	1.000	1.000	–							
4	EfficientNet-B0	0.212	0.535	0.212	–						
5	EfficientNet-B4	0.167	0.461	0.167	1.000	–					
6	EfficientNet-B7	0.029 *	0.130	0.029 *	0.999	1.000	–				
7	DenseNet121	0.999	1.000	0.999	0.683	0.610	0.212	–			
8	DenseNet169	0.461	0.812	0.461	1.000	1.000	0.980	0.907	–		
9	DenseNet201	0.610	0.907	0.610	1.000	0.999	0.940	0.964	1.000	–	
10	MobileNetV3Small	0.989	0.864	0.989	0.010 *	0.007 *	0.0005 *	0.751	0.040 *	0.074	–

**Table 5:** Nemenyi post hoc significant pairwise comparisons (p<0.05). * indicates statistically significant pairwise differences (p<0.05).

Comparison	*p*-Value
EfficientNet-B7 vs. MobileNetV3Small	0.0005 *
EfficientNet-B7 vs. ResNet18	0.0286 *
EfficientNet-B7 vs. ResNet152	0.0286 *
EfficientNet-B4 vs. MobileNetV3Small	0.0066 *
EfficientNet-B0 vs. MobileNetV3Small	0.0097 *
DenseNet169 vs. MobileNetV3Small	0.0398 *

## Data Availability

The data presented in this study are openly available in the Tufts Dental Database at https://tdd.ece.tufts.edu (accessed on 16 November 2025). The code used for training and evaluation is available from the corresponding author upon reasonable request.
